# Splenic NKG2D confers resilience versus susceptibility in mice after chronic social defeat stress: beneficial effects of (*R*)-ketamine

**DOI:** 10.1007/s00406-019-01092-z

**Published:** 2019-12-24

**Authors:** Kai Zhang, Akemi Sakamoto, Lijia Chang, Youge Qu, Siming Wang, Yaoyu Pu, Yunfei Tan, Xingming Wang, Yuko Fujita, Tamaki Ishima, Masahiko Hatano, Kenji Hashimoto

**Affiliations:** 1 Division of Clinical Neuroscience, Chiba University Center for Forensic Mental Health, 1-8-1 Inohana, Chiba, 260-8670 Japan; 2 Department of Biomedical Science, Chiba University Graduate School of Medicine, Chiba, 260-8670 Japan; 3 Present Address: Department of Psychiatry, Chaohu Hospital of Anhui Medical University, Hefei, 238000 China

**Keywords:** Antidepressant, Brain–spleen axis, (*R*)-ketamine, NKG2D, Spleen

## Abstract

The spleen is a large immune organ that plays a key role in the immune system. The precise molecular mechanisms underlying the relationship between the spleen and stress-related psychiatric disorders are unknown. Here we investigated the role of spleen in stress-related psychiatric disorders. FACS analysis was applied to determine the contribution of the spleen to susceptibility and resilience in mice that were subjected to chronic social defeat stress (CSDS). We found a notable increase in splenic volume and weight in CSDS-susceptible mice compared to control (no CSDS) mice and CSDS-resilient mice. The number of granulocytes, but not of T cells and B cells, in the spleen of susceptible mice was higher than in the spleen of both control and resilient mice. Interestingly, NKG2D (natural killer group 2, member D) expression in the spleen of CSDS-susceptible mice was higher than that in control mice and CSDS-resilient mice. In addition, NKG2D expression in the spleen of patients with depression was higher than that in controls. Both increased splenic weight and increased splenic NKG2D expression in CSDS-susceptible mice were ameliorated after a subsequent administration of (*R*)-ketamine. The present findings indicate a novel role of splenic NKG2D in stress susceptibility versus resilience in mice subjected to CSDS. Furthermore, abnormalities in splenic functions in CSDS-susceptible mice were ameliorated after subsequent injection of (*R*)-ketamine. Thus, the brain–spleen axis might, at least in part, contribute to the pathogenesis of stress-related psychiatric disorders such as depression.

**Electronic supplementary material:**

The online version of this article (10.1007/s00406-019-01092-z) contains supplementary material, which is available to authorized users.

## Introduction

Resilience is the ability to adapt successfully to stress and adversity. Stress resilience is mediated by adaptive changes in the central nervous system (CNS) and peripheral immune system, but the precise mechanisms behind stress susceptibility and resilience are unknown [1–7]. Mounting evidence indicates that the immune system plays a key role in stress-related psychiatric disorders such as major depressive disorder (MDD) [6–10]. Stress-induced inflammatory events in the CNS and peripheral immune system are linked with both susceptibility and resilience to stress [2, 7, 11]. Along with physiological and transcriptional adaptations of specific brain circuits, a crucial role in stress resilience is suggested for cellular and humoral factors of the immune system and changes at the interface between the brain and the periphery [7].

The spleen is the largest secondary immune organ, and it plays a key role in the development of stress-related psychiatric disorders [12–14]. Through a highly organized lymphoid compartment, the spleen can not only mount complex adaptive immune responses but also remove pathogens effectively from the blood [12]. It has been reported that chronic social defeat stress (CSDS), a rodent model of depression, increases the percentages of splenic CD11b^+^ myeloid cells, granulocytes, and CD11c^+^ dendritic cells and reduces the percentages of splenic natural killer (NK) cells [15, 16]. A recent study has shown that the spleen has a role in restraint stress-induced changes in the distribution of leukocytes in the blood [17]. Despite the effects of stress on the spleen being well-known, the precise molecular mechanisms underlying abnormalities in splenic functions caused by CSDS are still unknown. Moreover, there has been no report about the role of the spleen in stress susceptibility versus resilience in mice subjected to CSDS.

The aim of this study was to examine whether the spleen is involved in stress-related psychiatric disorders. Initially, we investigated the role of the spleen in stress susceptibility versus resilience in mice subjected to CSDS. Then, we studied the role of splenic NKG2D (Natural Killer Group 2, member D) in stress susceptibility versus resilience in mice subjected to CSDS, as NKG2D plays a vital role in the immune system [18–21]. Next, we measured NKG2D expression in the parietal cortex and the spleen from postmortem tissues from both controls and patients with major psychiatric disorders, including MDD, schizophrenia (SZ), and bipolar disorder (BD). The *N*-methyl-D-aspartate receptor antagonist (*R,S*)-ketamine is well-known to have robust antidepressant effects in treatment-resistant patients with depression [22–27]. Finally, we investigated whether a more potent enantiomer of (*R,S*)-ketamine, the rapid-acting and sustained antidepressant (*R*)-ketamine [25–34], can ameliorate abnormal splenic functions (i.e., increased volume and splenic functions) in CSDS-susceptible mice.

## Methods and materials

### Animals

Male adult C57BL/6 mice, aged 8 weeks (body weight 20–25 g, Japan SLC, Inc., Hamamatsu, Japan), and male adult CD1 mice, aged 14 weeks (body weight 40–45 g, Japan SLC, Inc., Hamamatsu, Japan) were used in the experiments. Animals were housed at a controlled temperature and under 12 h light/dark cycles (lights on between 07:00 and 19:00), with food and water ad libitum. The study was approved by the Chiba University Institutional Animal Care and Use Committee.

### Compounds and treatment

(*R*)-ketamine hydrochloride was prepared by recrystallizing (*R,S*)-ketamine and D-(−)-tartaric acid [28]. The dose (10 mg/kg as hydrochloride salt) of (*R*)-ketamine was selected as reported previously [29, 32–35]. Other reagents were purchased commercially.

### CSDS model

The CSDS procedure was performed as reported previously [29, 32–36]. Each day, for a total of 10 days, the C57BL/6 mice underwent 10 min exposure to a different CD1 aggressor mouse. At the end of the social defeat session, the resident CD1 mouse and the intruder mouse were housed in one half of the cage and separated by a perforated Plexiglas divider to enable visual, olfactory, and auditory contact for the remainder of the 24-h period. All mice were housed individually at 24 h after the final session. On day 11, we conducted a social interaction test (SIT) to identify subgroups of mice that were susceptible to and not susceptible to social defeat stress. This was achieved by placing mice in an interaction test box (42 cm × 42 cm), with an empty wire-mesh cage (10 cm × 4.5 cm) situated at one end. We tracked the movement of the mice 2.5 min, followed by a further 2.5 min in the presence of an unfamiliar aggressor confined in the wire-mesh cage. We used a stopwatch to record the length of time the subject spent in the “interaction zone” (defined as the 8-cm-wide area surrounding the wire-mesh cage). The interaction ratio was calculated as time spent in an interaction zone with an aggressor/time spent in an interaction zone without an aggressor. An interaction ratio of 1 was set as the cutoff: mice with scores < 1 were defined as “susceptible” to social defeat stress, whereas mice with scores ≥ 1 were defined as “resilient”. Approximately 70–80% of the mice were CSDS-susceptible. Susceptible mice were divided randomly in subsequent experiments involving administration of either saline or (*R*)-ketamine. Control mice were housed in the same cage before the behavioral tests.

### Antidepressant effects of (R,S)-ketamine and (R)-ketamine in a CSDS model

CSDS-susceptible mice were administered either saline (10 ml/kg) or (*R*)-ketamine (10 mg/kg) intraperitoneally (i.p.). Then, behavioral tests, including locomotion, a tail suspension test (TST), a forced swimming test (FST), and a one % sucrose preference test (SPT) were carried out as reported previously [29, 32–35, 37–39].

*Locomotion* Locomotor activity was measured using an animal movement analysis system SCANETMV-40 (MELQUEST Co., Ltd., Toyama, Japan). The animals were placed in experimental cages (length 560 mm × width 560 mm × height 330 mm). The cumulative exercise was recorded for 60 min. The ages were cleaned after each testing session.

*TST* We placed a small piece of adhesive tape approximately 2 cm from the tip of each mouse’s tail. We punched a single hole in the tape and hung the mice individually on hooks. Immobility was recorded over 10 min. Mice were regarded as immobile only when they hung passively and were completely motionless.

*FST* The FST was conducted using an automated forced-swim apparatus SCANETMV-40 (MELQUEST Co., Ltd., Toyama, Japan). The mice were placed individually in cylinders (diameter: 23 cm; height: 31 cm) containing 15 cm of water that was maintained at 23 ± 1 °C. The analysis software of the apparatus was used to record the immobility time from activity time as (total)–(active) time. The immobility time was recorded over 6 min.

*SPT* Mice were fed a water and 1% sucrose solution for 48 h, after which they underwent 4 h deprivation of water and food and 1 h exposure to two identical bottles, one containing water, and the other containing a 1% sucrose solution. These bottles were weighed both before and at the end of this period. The sucrose preference was calculated as the percentage of sucrose solution consumption to the total liquid consumption.

### Western blot analysis of NKG2D in mouse samples

The animals were killed by cervical dislocation and the spleens were removed rapidly. Half of each spleen was used for Western blot analysis and FACS analysis, respectively. The tissues were stored at − 80 °C prior to use. We performed Western blot analysis as reported previously [29, 34, 38, 39]. Tissue samples were homogenized in Laemmli lysis buffer. Aliquots (20 μg) of protein were measured using the DC protein assay kit (Bio-Rad) and incubated at 95 °C for 5 min, with an equal volume of 125 mM Tris–HCl, pH 6.8; 20% glycerol; 0.1% bromophenol blue; 10% β-mercaptoethanol and 4% SDS. The proteins were then subjected to SDS polyacrylamide gel electrophoresis using AnyKD minigels (Mini-PROTEAN TGX Precast Gel; Bio-Rad) and transferred onto PVDF membranes using a Trans Blot Mini Cell (Bio-Rad). For immunodetection, the blots were blocked with 2% BSA in TBST (TBS + 0.1% Tween-20) at room temperature for 1 h and kept with anti-NKG2D primary antibodies (1:1,000; Code No. ab203353: Abcam, Tokyo, Japan) at 4 °C overnight. The following day, blots were washed three times in TBST and incubated at room temperature for 1 h with horseradish peroxidase-conjugated anti-rabbit antibody (1:10,000). Following a final three washes with TBST, bands were detected using enhanced chemiluminescence (ECL) along with the Western Blotting Detection system (GE Healthcare Bioscience). The blots were then washed three times in TBST and incubated with the primary antibody directed against β-actin (1:10,000; Sigma-Aldrich). Images were captured using a Fuji LAS3000-mini imaging system (Fujifilm, Tokyo, Japan), and immunoreactive bands were quantified.

### Western blot analysis of NKG2D in human postmortem tissues

Human postmortem parietal cortex (Brodmann area 7) and spleen from both normal controls (*n* = 15) and MDD (*n* = 15), SZ (*n* = 15), and BD (*n* = 15) patients were obtained from the Stanley Foundation Brain Collection (Bethesda, MD) [40–43]. Spleen samples from three SZ patients and one MDD patient were not included [42]. Medical examiners collected the specimens, and permission was obtained from the next of kin in all cases. The demographic, clinical, and storage information for the cases had been published previously [40]. Each diagnostic group was matched based on several parameters, such as age at death, gender, postmortem interval, brain pH, and brain weight. This study was approved by the Research Ethics Committee of the Graduate School of Medicine, Chiba University. Western blot analysis of NKG2D in the parietal cortex and spleen was performed as described above.

### FACS analysis

Mouse spleen tissues were mashed and passed through a 70 μm mesh to prepare a single cell suspension and then subjected to FACS analysis. Spleen cells were suspended and their numbers were counted using an automated cell counter (BIO-RAD, Alfred Nobel Drive, CA). We stained 10^6^ cells with various monoclonal antibodies against cell surface antigens for 30 min at 4 °C and then washed them with an FACS buffer. For staining, we used the following antibodies: anti TCRb-FITC (cat# 553170: BD Bioscience, Franklin Lakes, NJ), anti-B220-PE (cat# 553090: BD Bioscience), anti-NKG2D- allophycocyanin (cat# 17-5882-82: eBioscience), anti-Gr1-PE (cat# 55161: BD Bioscience), and anti-DX5-PE (cat# 553,858: BD Bioscience). The stained cells were analyzed using FACSCantII and FlowJo software (BD Bioscience).

### Statistical analysis

The data are shown as the mean ± standard error of the mean (S.E.M.). Data were analyzed using PASW Statistics 20 (formerly SPSS Statistics; SPSS) and one-way analysis of variance, followed by a *post-hoc* Tukey test. *P *values < 0.05 were considered statistically significant.

## Results

### FACS analysis of spleen samples

**Fig. 1:**
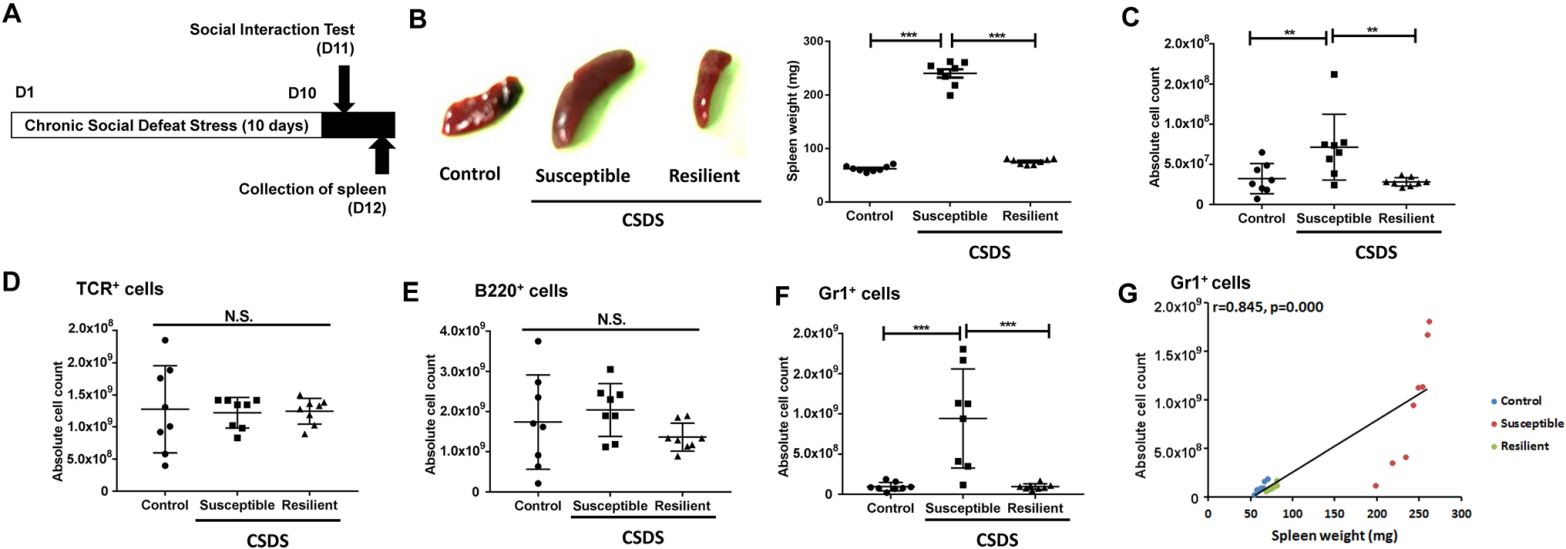
Schedule of CSDS and FACS analysis. **a** The schedule of chronic social defeat stress (CSDS) model, social interaction test, and collection of spleen. CSDS was performed for 10 days (day 1- day 10). On day 11, the social interaction test was performed to separate CSDS-susceptible mice and CSDS-resilient mice. Control (no CSDS) mice were used as control group. On day 12, spleen from the three groups was collected. **b** Representative picture of spleen from control (no CSDS) mice, CSDS-susceptible mice, and CSDS-resilient mice. Spleen weight of CSDS-susceptible mice was significantly higher than that of control mice and CSDS-resilient mice (one-way ANOVA, *F*_2,21_ = 437.136, *P* < 0.001). **c** Total cell number of spleen (one-way ANOVA, *F*_2,21_ = 6.616, *P* = 0.006). **d** T cells: The number of TCR^+^ cells in the spleen (one-way ANOVA, *F*_2,21_ = 0.032, *P* = 0.968). **e** B cells: The number of B220^+^ in the spleen (one-way ANOVA, *F*_2,21_ = 1.429, *P* = 0.262). **f** Granulocyte: The number of Gr1^+^ cells in the spleen (one-way ANOVA, *F*_2,21_ = 14.958, *P* < 0.001). **g** A positive correlation (r = 0.845, *P* < 0.001) between Gr1^+^ cells (for granulocytes) and spleen weight among three groups. Data are shown as mean ± SEM. (*n* = 8). **P < 0.01, ***P < 0.001 *ANOVA* analysis of variance, *B220* B cells, *Gr1* granulocyte receptor 1, *CD11b* a marker of macrophages, *N.S.* not significant, *TCR* T cell receptor

Spleen samples were collected from control mice (no CSDS), CSDS-susceptible mice, and CSDS-resilient mice (Fig. 1a). Unexpectedly, the spleens in the CSDS-susceptible mice were significantly larger and heavier than those of both control mice and CSDS-resilient mice (Fig. 1b). We used FACS analysis to count the numbers of T cells, B cells, and granulocytes in the spleens from the three groups. The total cell numbers from the spleens of CSDS-susceptible mice were significantly higher than were those from the spleens of both control mice and CSDS-resilient mice (Fig. 1c). Conversely, the numbers of T cells and B cells were similar in the three groups (Fig. 1d, e). Further, the numbers of Gr1^+^ granulocytes in the spleens of CSDS-susceptible mice were significantly higher than in both control mice and CSDS-resilient mice (Fig. 1f). Interestingly, there were positive correlations between the number of Gr1^+^ cells and spleen weight in all groups (Fig. 1g). Collectively, it is probable that the increased spleen weight in CSDS-susceptible mice might be due to the spleens in these animals having a greater number of granulocytes.

### Relationship between depression-like phenotypes and splenic NKG2D expression

**Fig. 2:**
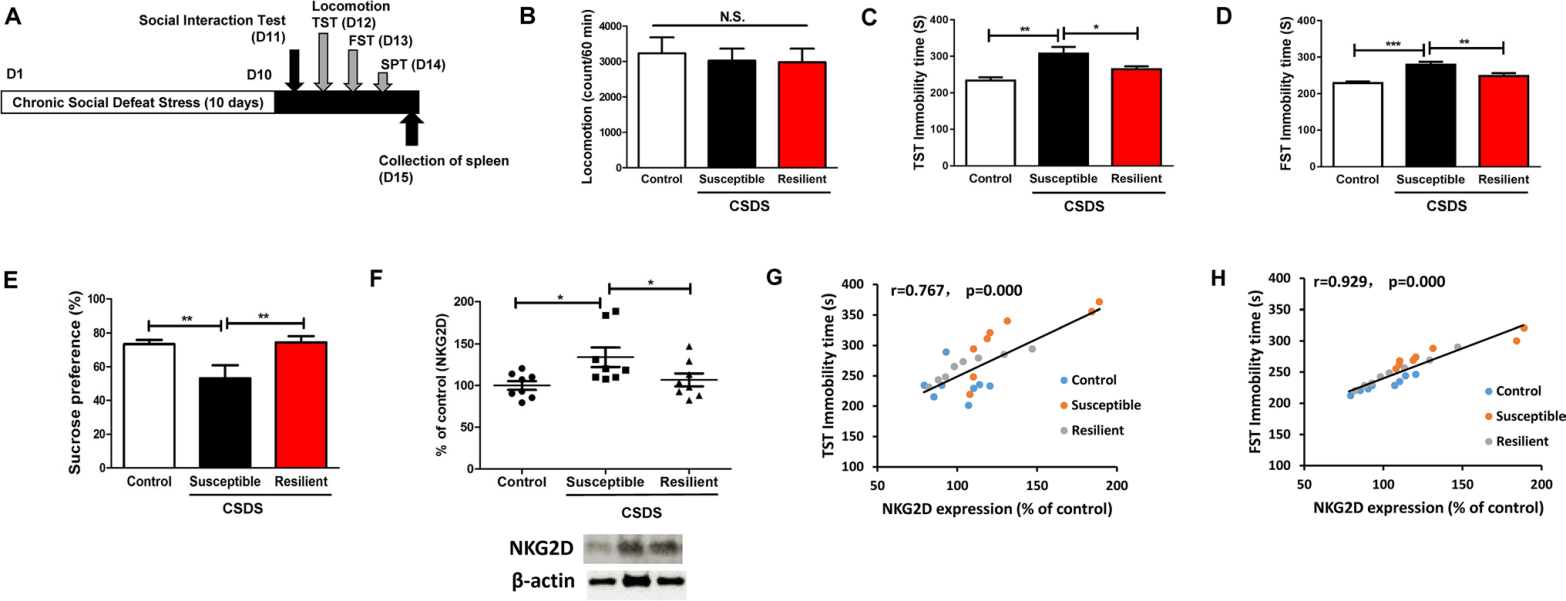
Behavioral tests and NKG2D expression in the spleen of CSDS-susceptible mice and CSDS-resilient mice. **a** The schedule of CSDS model, social interaction test, behavioral tests, and collection of spleen. CSDS was performed for 10 days (day 1–day 10). On day 11, the social interaction test was performed to separate CSDS-susceptible mice and CSDS-resilient mice. On day 12, locomotion and TST were performed. On day 13, FST was performed. On day 14, SPT was performed. On day 15, spleen from the three groups was collected. Control (no CSDS) mice were used as control group. **b** Locomotion (one-way ANOVA, *F*_2,21_ = 0.118, *P* = 0.889). **c** TST (one-way ANOVA, *F*_2,21_ = 8.494, *P* = 0.002). **d** FST (one-way ANOVA, *F*_2,21_ = 13.317, *P* < 0.001). **e** SPT (one-way ANOVA, *F*_2,21_ = 5.395, *P* = 0.013). **f** NKG2D expression in the spleen (one-way ANOVA, *F*_2,21_ = 4.278, *P* = 0.028). **g** A positive correlation (r = 0.767, *P* < 0.001) between TST immobility time and NKG2D expression in the spleen. **h** A positive correlation (r = 0.929, *P* < 0.001) between FST immobility time and NKG2D expression in the spleen. Data are shown as mean ± SEM. (*n* = 8). *P < 0.05, **P < 0.01, ***P < 0.001. *ANOVA* analysis of variance, *FST* forced swimming test, *N.S.* not significant, *SPT* sucrose preference test, *TST* tail suspension test

After CSDS, CSDS-susceptible mice and CSDS-resilient mice were divided by means of a SIT (Fig. 2a). Then, we conducted behavioral tests, including locomotion, TST, FST, and SPT (Fig. 2a). There were no changes in locomotion in the three groups (Fig. 2b). The immobility time of TST and FST in the CSDS-susceptible mice was significantly higher than in both control mice and CSDS-resilient mice (Fig. 2c, d). Moreover, in CSDS-susceptible mice the sucrose preference of SPT was significantly lower than was the case in both control mice and CSDS-resilient mice (Fig. 2e).

NKG2D is a lectin-like type 2 transmembrane stimulatory immunoreceptor that is expressed by all NK cells and by subsets of T cells [18–21]. Because of the key contribution of NKG2D in the immune system [18–20, 44], we examined the role of NKG2D in the spleen in a CSDS model. Western blot analysis revealed that NKG2D expression in the spleen of CSDS-susceptible mice was significantly higher than that in both control mice and CSDS-resilient mice (Fig. 2f). Interestingly, there were positive correlations between the immobility time of TST (or FST) and NKG2D expression in the spleen in the three groups (Fig. 2g, h). These findings indicate that splenic NKG2D may play a role in depression-like phenotypes in a CSDS model.

### Expression of NKG2D protein in the postmortem tissues (parietal cortex and spleen) from patients with MDD, SZ, and BD

**Fig. 3:**
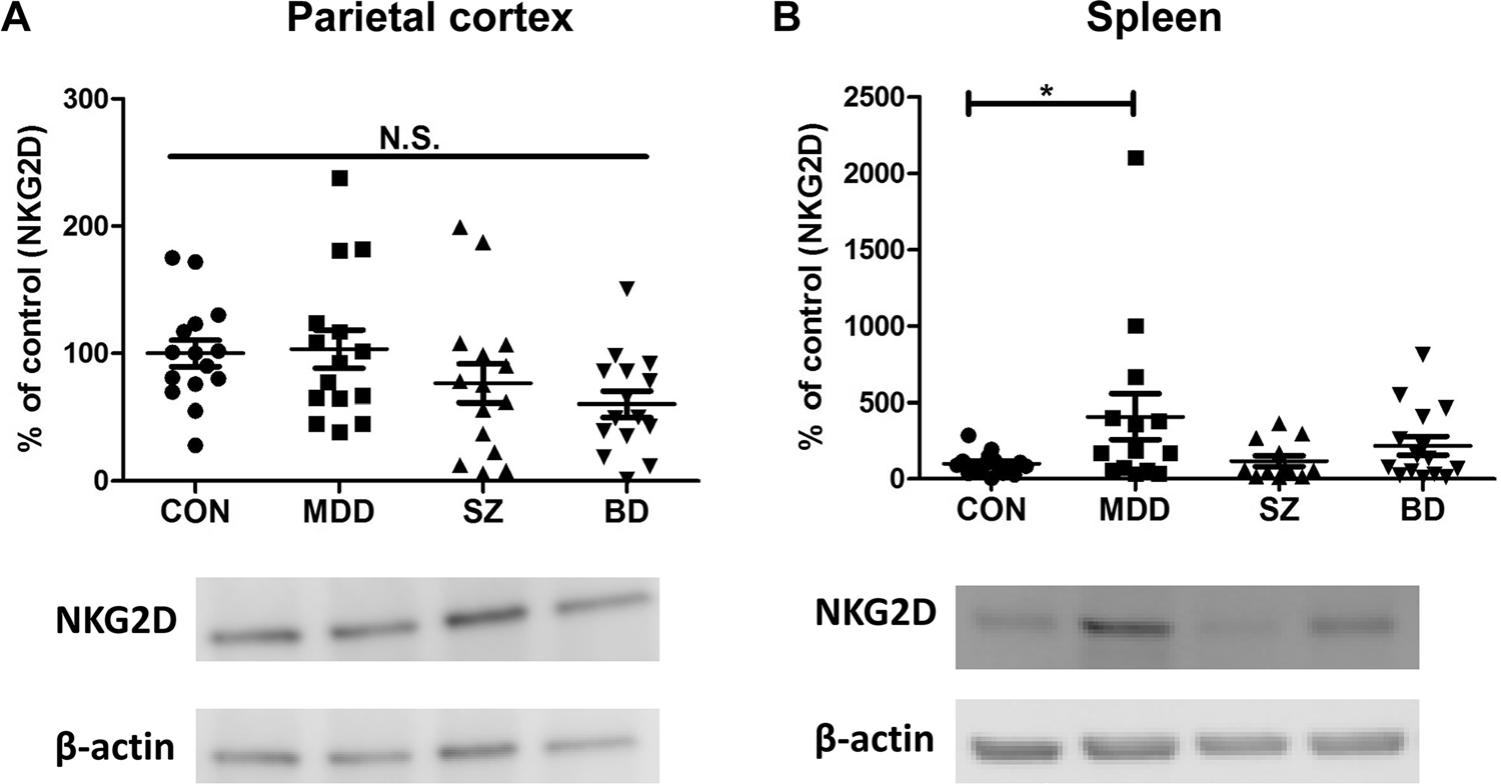
Protein expression of NKG2D in the parietal cortex and spleen from patients with major psychiatric disorders. **a** Parietal cortex: There were no changes (one-way ANOVA, *F*_3,53_ = 3.004, *P* = 0.051) among the four groups. The bands of representative bands of Western blot analysis were shown. Data are shown as mean ± SEM. (*n* = 15). **b** Spleen: There were significant changes (one-way ANOVA, *F*_3,52_ = 2.673, *P* = 0.048) among the four groups. Expression of NKG2D in the spleen of MDD patients was significantly (*P* = 0.012) higher than controls. *P < 0.05. Data are shown as mean ± SEM. (*n* = 12–15). The bands of representative bands of Western blot analysis were shown. *CON* control, *MDD* major depressive disorder, *SZ* schizophrenia, *BD* bipolar disorder, *N.S.* not significant

We conducted Western blot analysis of NKG2D protein in the parietal cortex and spleen from patients with MDD, SZ, or BD. There were no changes in NKG2D expression in the parietal cortex in the four groups (Fig. 3a). Conversely, there were significant changes in NKG2D expression in the spleen in the four groups (Fig. 3b). Interestingly, NKG2D expression in the spleen was significantly higher in MDD patients than in controls (Fig. 3b). These findings indicate that increased NKG2D expression in the spleen of MDD patients might contribute to the pathogenesis of MDD.

### Effects of (R)-ketamine in the splenic functions of CSDS-susceptible mice

**Fig. 4:**
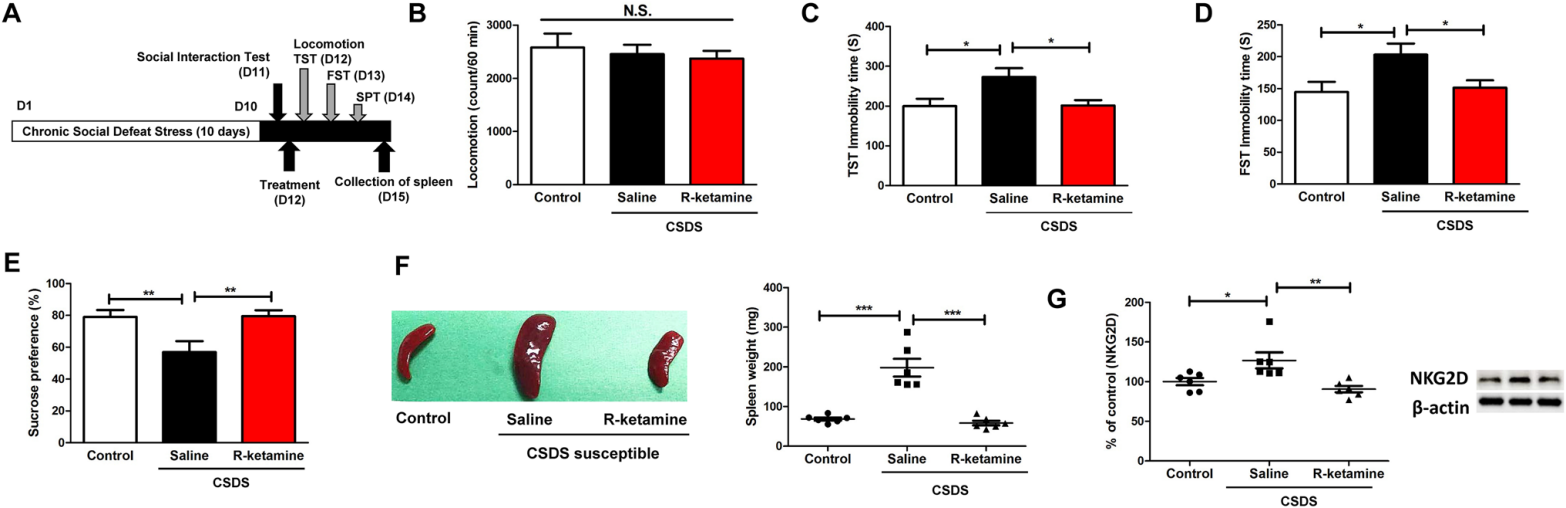
Effects of (*R*)-ketamine on depression-like phenotypes, abnormal splenic functions in the CSDS-susceptible mice. **a** The schedule of CSDS model, social interaction test, treatment, behavioral tests, and collection of spleen. CSDS was performed for 10 days (day 1–day 10). On day 11, the social interaction test was performed to select CSDS-susceptible mice. Control (no CSDS) mice were used as control group. On day 12, saline (10 ml/kg) or (*R*)-ketamine (10 mg/kg) was administered i.p. into CSDS-susceptible mice. Saline was administered i.p. into control (no CSDS) mice. Locomotion and TST were performed 1 and 3 h after injection, respectively. On day 13, FST was performed. On day 14, SPT was performed. On day 15, spleen from the three groups was collected. **b** Locomotion (one-way ANOVA, *F*_2,15_ = 0.270, *P* = 0.767). **c** TST (one-way ANOVA, *F*_2,15_ = 5.188, *P* = 0.019). **d** FST (one-way ANOVA, *F*_2,15_ = 4.446, *P* = 0.030). **e** SPT (one-way ANOVA, *F*_2,15_ = 6.376, *P* = 0.010). **f** Representative picture of spleen from saline-treated control (no CSDS) mice, saline-treated CSDS-susceptible mice, and (*R*)-ketamine-treated CSDS-susceptible mice. Spleen weight of (*R*)-ketamine-treated CSDS-susceptible mice was significantly lower higher than that of saline-treated CSDS-resilient mice (one-way ANOVA, *F*_2,15_ = 12.052, *P* = 0.001). **g** Western blot analysis of NKG2D expression in the spleen (one-way ANOVA, *F*_2,15_ = 7.591, *P* = 0.005). The representative bands of Western blot analysis were shown. Data are shown as mean ± SEM. (*n* = 6). *P < 0.05, **P < 0.01, ***P < 0.001. *ANOVA* analysis of variance, *ANOVA* analysis of variance, *FST* forced swimming test, *NKG2D* natural-killer receptor group 2, member D, *N.S.* not significant, *SPT* sucrose preference test, *TST* tail suspension test

Previously, using rodent models of depression, we reported that (*R*)-ketamine has greater and longer-lasting antidepressant effects than does the US FDA-approved antidepressant (*S*)-ketamine [25–35]. Therefore, we examined whether (*R*)-ketamine (10 mg/kg) can ameliorate abnormal splenic functions in CSDS-susceptible mice (Fig. 4a). There were no changes in locomotion in the three groups (Fig. 4b). Compared to saline, (*R*)-ketamine significantly attenuated the increased immobility time of TST and FST in the CSDS-susceptible mice (Fig. 4c, d). Moreover, compared to saline, (*R*)-ketamine significantly enhanced the decreased sucrose preference of SPT in the CSDS-susceptible mice (Fig. 4e). Interestingly, a single injection of (*R*)-ketamine significantly ameliorated the increased size and weight of the spleen in CSDS-susceptible mice (Fig. 4f). Moreover, compared to saline, (*R*)-ketamine significantly attenuated the increased splenic NKG2D expression in CSDS-susceptible mice (Fig. 4g). Interestingly, in all groups, there were positive correlations between the immobility time of TST (or FST) and spleen weight (Figures S1A and S1B). Conversely, in all groups, there was a negative correlation between sucrose preference of SPT and spleen weight (Figure S1C). Moreover, in all groups, there was a positive correlation between splenic NKG2D expression and spleen weight (Figure S1D). These findings indicate that splenic NKG2D might contribute to depression-like phenotypes in CSDS-susceptible mice.

**Fig. 5:**
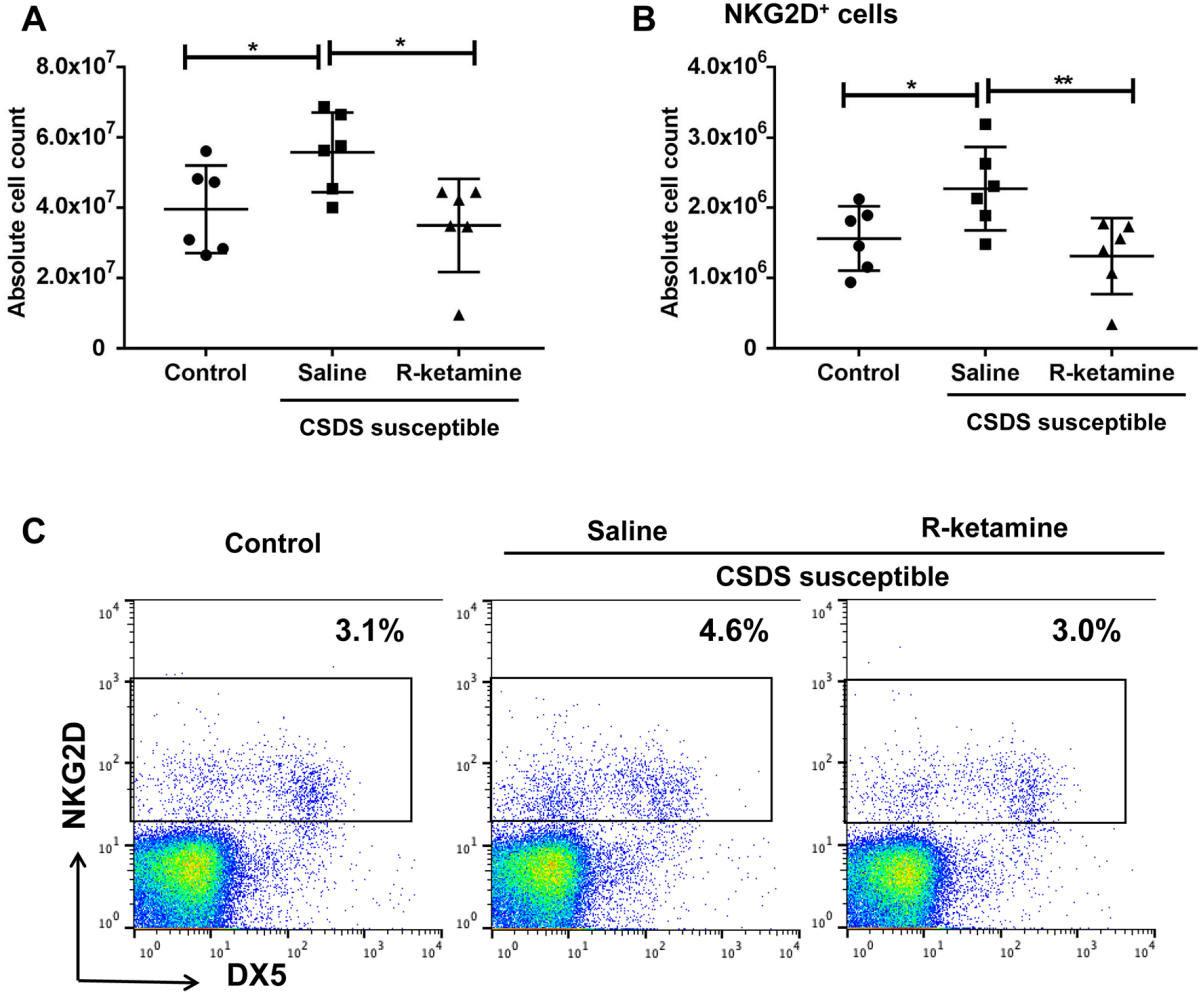
FACS analysis of spleen samples of three groups. **a** Total cell number of spleen (one-way ANOVA, *F*_2,15_ = 4.659, *P* = 0.027). **b** The number of NKG2D^+^ cells in the spleen (one-way ANOVA, *F*_2,15_ = 5.207, *P* = 0.019). Data are shown as mean ± SEM. (*n* = 6). *P < 0.05, **P < 0.01. **c** FACS analysis of cells stained with antibodies against NKG2D and DX5 in spleen samples from saline-treated control mice, saline-treated CSDS-susceptible mice, and (*R*)-ketamine-treated CSDS-susceptible mice. (*R*)-ketamine ameliorated the increased percentage of NKG2D^+^ cells in the spleen of CSDS-susceptible mice. *ANOVA* analysis of variance, *DX5* CD49b, *NKG2D* natural-killer receptor group 2, member D

Subsequently, we conducted FACS analysis of spleen samples from the three groups. Compared to saline, (*R*)-ketamine (10 mg/kg, 3 days after injection) attenuated significantly the increased spleen cell number of CSDS-susceptible mice (Fig. 5a). Furthermore, compared to saline, (*R*)-ketamine attenuated significantly the increased number of NKG2D^+^ cells in the spleen of CSDS-susceptible mice (Fig. 5b, c). There were also positive correlations between the immobility time of TST and FST (but not anhedonia) and splenic NKG2D expression in all groups (Figure S2A–S2C).

## Discussion

**Fig. 6:**
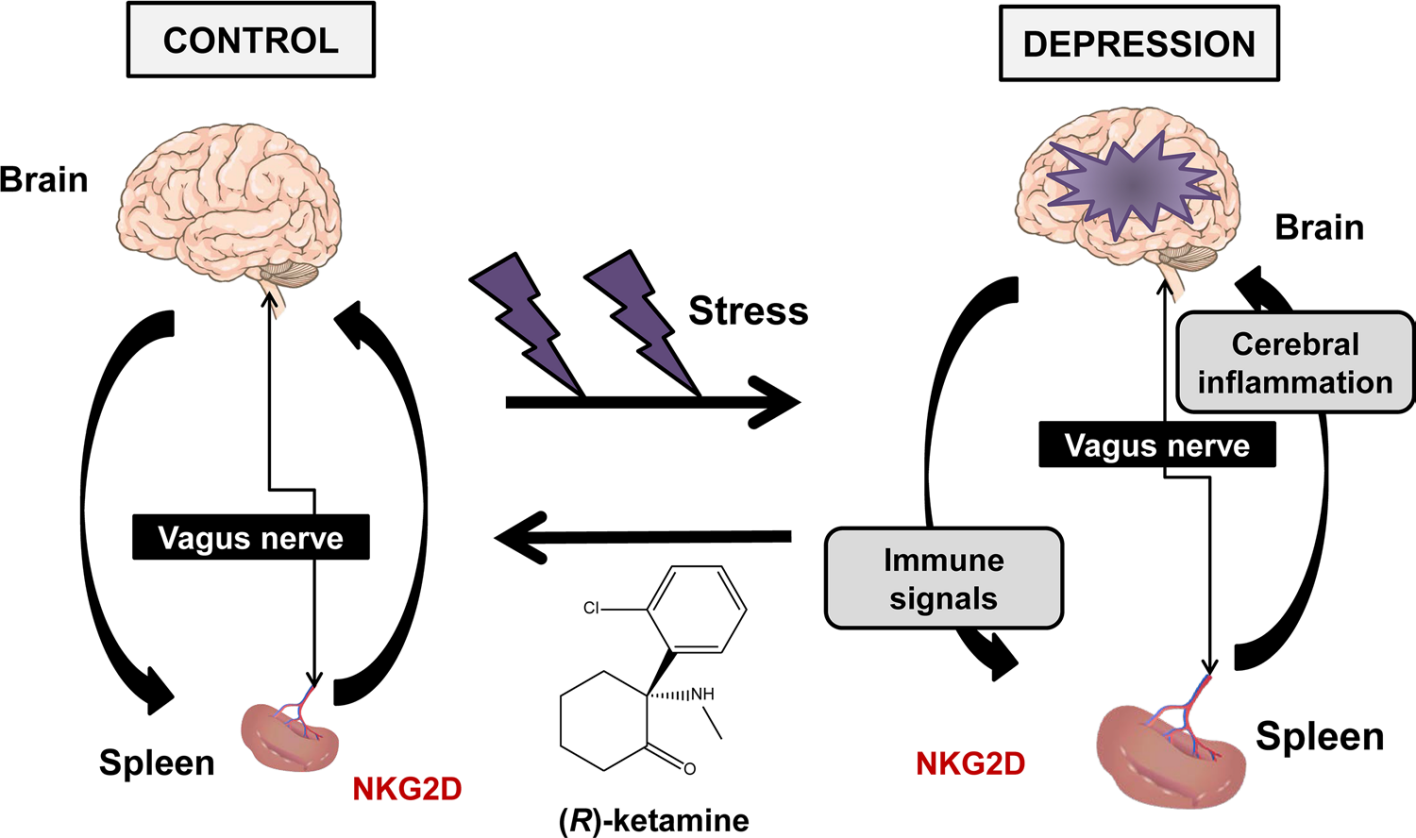
Proposed mechanisms of the role of brain–spleen axis in the stress-induced psychiatric disorders. Stress caused increase of spleen size and weight, resulting in abnormalities in spleen functions. Brain inflammation by stress might be mediated by brain–spleen axis (i.e., vagus nerve). NKG2D in spleen may play a role in the stress-related psychiatric disorders such as depression. Interestingly, (*R*)-ketamine ameliorates abnormalities of spleen functions and depressive symptoms by stress

The main findings of this study are as follows: first, in CSDS-susceptible mice, the spleen was larger and heavier than in both control (no CSDS) mice and CSDS-resilient mice. FACS analysis of spleen revealed that there were more granulocytes, but not T cells and B cells, in the CSDS-susceptible mice than in both control mice and CSDS-resilient mice. Interestingly, there were positive correlations between the number of granulocytes in the spleen and spleen weight. Thus, it appears that increased size and weight of the spleen in CSDS-susceptible mice might result from the increase in the number of granulocytes in the spleen of these mice. Second, NKG2D expression in the spleen of CSDS-susceptible mice was higher than that in both control mice and CSDS-resilient mice. Interestingly, there were positive correlations between the immobility time of TST (or FST) and splenic NKG2D expression in the three groups. It appears that increased expression of NKG2D in the spleen may contribute to the depression-like phenotypes in CSDS-susceptible mice. Importantly, we found increased NKG2D expression in the spleen from MDD patients compared to controls, whereas there were no changes in the parietal cortex of MDD patients. Third, in the CSDS-susceptible mice, a single injection of (*R*)-ketamine had fast-acting antidepressant effects, consistent with our previous reports [29, 32–35]. Interestingly, three days after a single injection of (*R*)-ketamine, the increased size and weight of spleen in CSDS-susceptible mice recovered to control levels. Moreover, in these mice, there were correlations between the severity of depression-like phenotypes and spleen weight. (*R*)-ketamine attenuated significantly the increased splenic NKG2D expression in the CSDS-susceptible mice. Moreover, (*R*)-ketamine attenuated significantly the increases in total cell number and NKG2D^+^ cells in the spleen of CSDS-susceptible mice. These findings indicate that splenic NKG2D confers resilience versus susceptibility in mice subjected to CSDS. Moreover, (*R*)-ketamine might, in part, exert antidepressant-like effects by normalizing splenic NKG2D in CSDS-susceptible mice. Finally, the brain–spleen axis may well, at least in part, play a role in stress-related psychiatric disorders (Fig. 6).

In 2018, McKim et al. [45] reported that the total number of cells within the spleen was increased ~ 2.6-fold after modified CSDS and that modified CSDS-induced numbers of erythrocytes, monocytes, and granulocytes within the spleen are associated with a ~ twofold increase in spleen size and weight. However, they did not use a social interaction to divide susceptible mice and resilient mice, suggesting that they used both susceptible and resilient mice subjected to modified CSDS [45]. Notably, in the CSDS-resilient mice, spleen size and weight, in addition to splenic functions, were similar to those in control (no CSDS) mice. Collectively, it is likely that CSDS-induced alterations in splenic functions (i.e., granulocytes) confer stress susceptibility versus resilience in mice after CSDS, although there is a need for further detailed study on the spleen’s role in stress resilience. Further, it is of great interest to study whether spleen size is changed in medication-free patients with stress-related disorders such as MDD.

NKG2D is a potent activator of the immune system that translates cellular stress into activation signals for immune cells [21, 44, 46]. Accumulating data support the role of inflammation- and stress-induced expression of NKG2D ligands in several immune-mediated diseases [21], indicating that NKG2D can be a therapeutic target for inflammation-related diseases. In this study, we found increased NKG2D expression in the spleen of CSDS-susceptible mice compared to both control and CSDS-resilient mice. We also found positive correlations between the immobility time of TST (or FST) and splenic NKG2D expression in control, susceptible, and resilient groups. It should be noted that the NKG2D expression in the spleen of CSDS-resilient mice was similar to that in control (no CSDS) mice. A study using NKG2D-deficient mice showed that there were fewer cells in the spleen of NKG2D-deficient mice than in the spleen of age-matched control mice, and that deficient mice were more resistant to murine cytomegalovirus infection [47]. Collectively, CSDS-induced increases in splenic NKG2D likely confer stress susceptibility versus resilience in mice after CSDS, although there is need for further study of the role of splenic NKG2D in stress resilience. Nonetheless, it is very interesting to study the role of splenic NKG2D in stress resilience versus susceptibility using spleen-specific NKG2D-deficient mice.

In this study, we also found positive correlations between the immobility time of TST (or FST) and NKG2D expression in the spleen in control, saline-treated susceptible, and (*R*)-ketamine-treated susceptible groups. Moreover, there was positive correlation between splenic NKG2D expression and spleen weight in the three groups, indicating that NKG2D may be involved in spleen function. Collectively, it is likely that increased splenic NKG2D expression may contribute both to the depression-like phenotypes and to increased spleen size and weight in CSDS-susceptible mice. It should be noted that a single injection of (*R*)-ketamine ameliorated the increased splenic NKG2D expression in CSDS-susceptible mice. It is of great interest to study whether (*R*)-ketamine can affect spleen size in patients with stress-related psychiatric disorders, as a clinical trial of (*R*)-ketamine is underway [26]. Additionally, (*R*)-ketamine might be a potential therapeutic drug for inflammation-related diseases, as a clinical trial of anti-NKG2D antibody is underway for inflammation-related diseases such as Crohn’s disease and rheumatoid arthritis [21, 48].

Multiple lines of evidence indicate that abnormal composition of gut microbiota may contribute to resilience versus susceptibility in rodents after either CSDS or inescapable electric stress [7, 49–54]. Further study of the role of brain-gut microbiota and spleen in stress susceptibility and resilience is also of interest (Fig. 6).

In conclusion, this study reveals that increased size and weight of the spleen in CSDS-susceptible mice might be associated with increases in the number of granulocytes in the spleen, and that splenic NKG2D might be associated with susceptibility versus resilience in mice following CSDS. Moreover, (*R*)-ketamine ameliorated abnormal splenic functions (i.e., increases in spleen size and NKG2D expression) in CSDS-susceptible mice. Finally, we propose a new hypothesis that the brain–spleen axis might, at least in part, play a role in stress-related psychiatric disorders.

## Electronic supplementary material

Below is the link to the electronic supplementary material.

Supplementary file1 (DOCX 211 kb)
