# Supplementary material for: Splenic NKG2D confers resilience versus susceptibility in mice after chronic social defeat stress: beneficial effects of (R)-ketamine
Source: Eur Arch Psychiatry Clin Neurosci. 2019 Dec 24;271(3):447–56. doi: 10.1007/s00406-019-01092-z (PMC7981328; doi:10.1007/s00406-019-01092-z)
Supplement: Supplementary file 1 — Supplementary file1 (DOCX 211 kb) [file 406_2019_1092_MOESM1_ESM.docx]

**Supplemental Information**

**Splenic NKG2D confers resilience versus susceptibility in mice after chronic social defeat stress: Beneficial effects of (*R*)-ketamine**

Kai Zhang, Akemi Sakamoto, Lijia Chang, Youge Qu, Siming Wang, Yaoyu Pu, Yunfei Tan, Xingming Wang, Yuko Fujita, Tamaki Ishima, Masahiko Hatano, and Kenji Hashimoto

Division of Clinical Neuroscience (KZ, LC, YF, YQ, SW, YP, YT, XW, TI, YS, KH), Chiba University Center for Forensic Mental Health, Chiba 260-8670, Japan; Department of Biomedical Science (AS, MH), Chiba University Graduate School of Medicine, Chiba 260-8670, Japan.

**Correspondence:** Dr. Kenji Hashimoto. Division of Clinical Neuroscience, Chiba University Center for Forensic Mental Health, 1-8-1 Inohana, Chiba 260-8670, Japan Telephone number: +81-43-226-2517, E-mail: hashimoto@faculty.chiba-u.jp

Current position of Dr. Kai Zhang: Department of Psychiatry, Chaohu Hospital of Anhui Medical University, Hefei 238000, China.

**Short title:** Splenic NKG2D in stress resilience and susceptibility


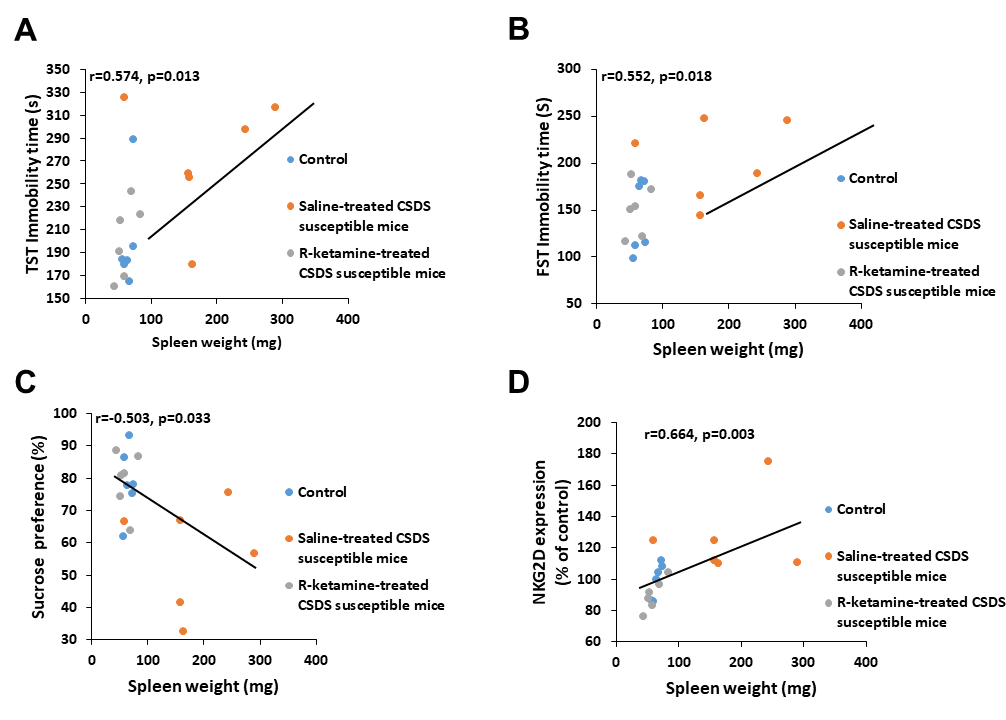


**Figure S1. Correlations between depression-like phenotypes (or NKG2D and spleen weight**

(A): A positive correlation (r = 0.574, P = 0.013) between TST immobility time and spleen weight among three groups. (B): A positive correlation (r = 0.552, P = 0.018) between FST immobility time and spleen weight among three groups. (C): A negative correlation (r = - 0.503, P = 0.033) between sucrose preference and spleen weight among three groups. (D): A positive correlation (r = 0.664, P = 0.003) between NKG2D expression and spleen weight among three groups.

**
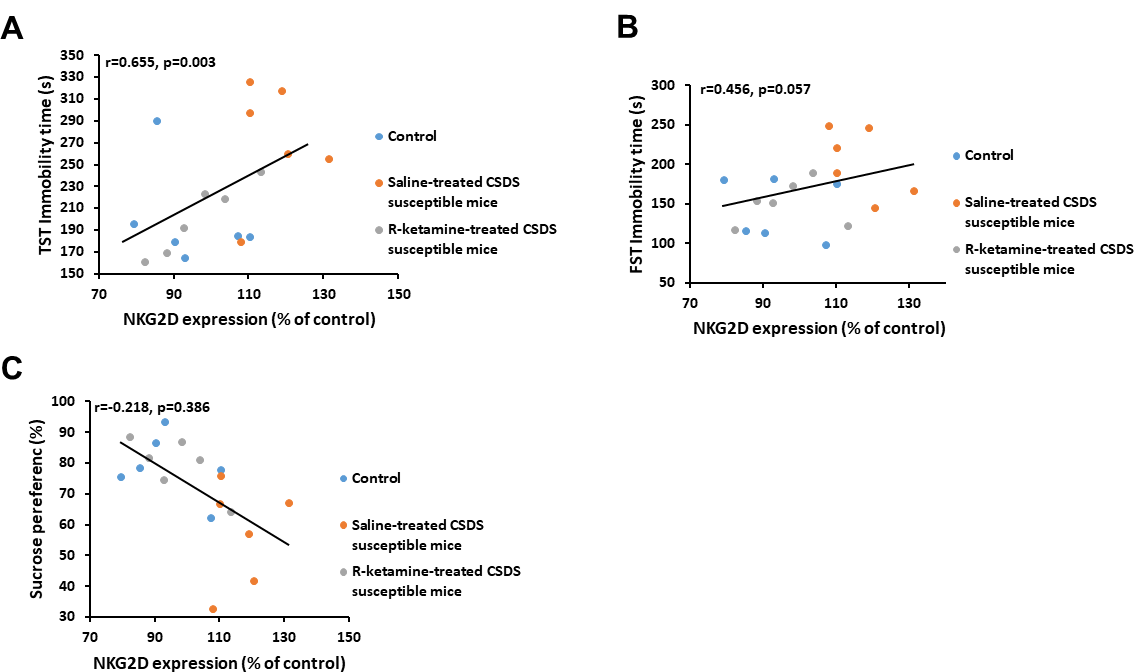
**

**Figure S2. Correlations between depression-like phenotypes and NKG2D expression**

(A): A positive correlation (r = 0.574, P = 0.013) between TST immobility time and spleen weight among three groups. (B): A positive correlation (r = 0.552, P = 0.018) between FST immobility time and spleen weight among three groups. (C): A negative correlation (r = - 0.503, P = 0.033) between sucrose preference and spleen weight among three groups. (D): A positive correlation (r = 0.664, P = 0.003) between NKG2D expression and spleen weight among three groups.
